# An Implementation Science Laboratory as One Approach to Whole System Improvement: A Canadian Healthcare Perspective

**DOI:** 10.3390/ijerph182312681

**Published:** 2021-12-01

**Authors:** Rachel Flynn, Stephanie P. Brooks, Denise Thomson, Gabrielle L. Zimmermann, David Johnson, Tracy Wasylak

**Affiliations:** 1 Faculty of Nursing, University of Alberta, Edmonton, AB T6G 1C9, Canada; 2 Alberta SPOR SUPPORT Unit, Faculty of Medicine and Dentistry, University of Alberta, Edmonton, AB T6G 2C8, Canada; stephaniebrooks@ualberta.ca (S.P.B.); dthomson@ualberta.ca (D.T.); gzimmerm@ualberta.ca (G.L.Z.); david.johnson@albertahealthservices.ca (D.J.); tracy.wasylak@ahs.ca (T.W.); 3 Department of Community Health Sciences, Cumming School of Medicine, University of Calgary, Calgary, AB T2N 4Z6, Canada; 4 Alberta Health Services, Calgary, AB T2N 1S7, Canada; 5 Faculty of Nursing, University of Calgary, Calgary, AB T2N 4Z6, Canada

**Keywords:** implementation science, implementation practice, whole system, improvement, learning health system

## Abstract

Implementation science (IS) has emerged as an integral component for evidence-based whole system improvement. IS studies the best methods to promote the systematic uptake of evidence-based interventions into routine practice to improve the quality and effectiveness of health service delivery and patient care. IS laboratories (IS labs) are one mechanism to integrate implementation science as an evidence-based approach to whole system improvement and to support a learning health system. This paper aims to examine if IS labs are a suitable approach to whole system improvement. We retrospectively analyzed an existing IS lab (Alberta, Canada’s Implementation Science Collaborative) to assess the potential of IS labs to perform as a whole system approach to improvement and to identify key activities and considerations for designing IS labs specifically to support learning health systems. Results from our evaluation show the extent to which IS labs support learning health systems through enabling infrastructures for system-wide improvement and research.

## 1. Introduction

Fewer than 40% of healthcare improvement innovations successfully transition from adoption to sustained implementation that spreads to more than one area of an organization [1,2]. Achieving whole system improvement is often hindered by innovations themselves [3,4], how they are implemented [5,6], the characteristics of the individuals involved [7,8], organizational factors [9,10], and features of the wider environment [11].

Learning health systems are gaining momentum as frameworks to coordinate health services and research to overcome common improvement barriers and enable increased value and quality of care on a whole system level. Learning health systems are dynamic ecosystems where scientific, social, technological, policy, legal, and ethical dimensions are aligned to enable continuous learning and improvement to be embedded across the system [12]. Achieving a learning health system requires robust, integrated implementation science theory, evidence and skilled expertise, comprehensive data, standardized approaches to implementation and evaluation, a supportive change culture, collaborative networks, and stakeholder involvement [12,13]. Specifically, the goal of a learning health system is to improve patient experiences, improve provider experiences, improve health outcomes, and decrease health care costs [14].

The continuous knowledge creation in learning health systems necessitates ongoing implementation, spread, scale, and sustainment of new innovations. Successful uptake of innovations into practice requires the consideration of multiple health system levels, carefully planned implementation strategies, and the ability to adapt elements of evidence-based interventions to fit context [15]. The scientific discipline of implementation science (IS) studies the best methods to promote the systematic uptake of innovations into routine practice, to improve the quality and effectiveness of health service delivery and patient care [16]. Globally, a number of reviews and policies identify the need to integrate implementation practice and science to drive healthcare improvement at a system level [17,18,19,20]. However, to date, there has been limited convergence between these fields.

Implementation science laboratories (IS labs) have emerged as one approach to enable the integration of implementation science teams into health systems [21]. IS labs consist of multi-disciplinary teams of applied scientists from academia (e.g., implementation, behaviour, data researchers, and health economists) that work in partnership with health system stakeholders (i.e., health system innovation teams, decision makers, leaders, implementation support practitioners, change management teams, and frontline staff) to co-produce theory-based implementation and evaluation support for innovations [21]. Some of the published literature suggests the potential for IS labs to support healthcare improvement beyond specific innovation projects to enable a learning health system [22,23]. However, the role of IS labs within learning health systems has not been well characterized.

### 1.1. Research Aim

Our research aimed to evaluate the alignment of one IS lab, Alberta, Canada’s Implementation Science Collaborative (ISC), with an established learning health system conceptual framework [12] to (a) assess the potential of IS labs to perform as a whole system approach to improvement and (b) identify key activities and considerations for designing IS labs specifically to support learning health systems. Understanding these aims is critical for the next stages of the ISC’s implementation.

### 1.2. Research Context

The ISC is situated within the context of Alberta Health Services, Alberta’s province-wide, publicly funded health system in Canada that serves a population of 4.3 million people [24]. For over a decade, Alberta Health Services has been working to transform into a learning health system [25,26,27]. The ISC is one initiative designed to enable and accelerate Alberta’s existing learning health system.

**Figure 1:**
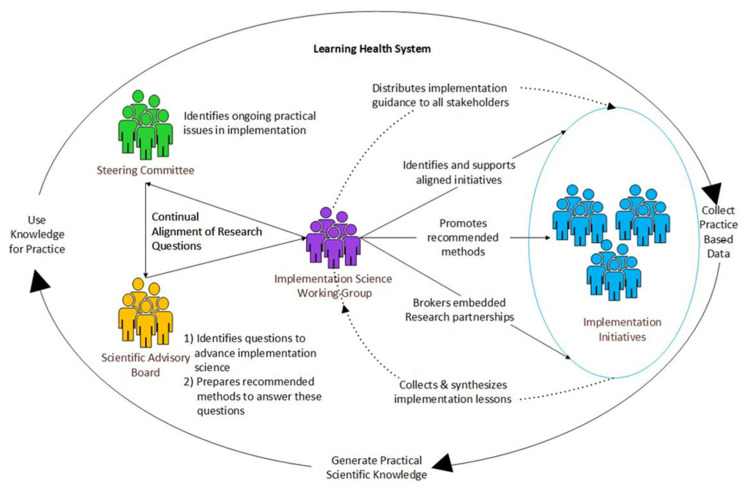
The ISC model.

The ISC is led by the Alberta Strategy for Patient-Oriented Research SUPPORT Unit (AbSPORU), which is part of Canada’s Strategy for Patient-Oriented Research, a national coalition of federal, provincial, and territorial partners (including patients and informal caregivers, provincial health authorities, and academic health centres) dedicated to the integration of research into care [28]. A co-design approach [29] between health system decision makers, operations, academic institutions, government authorities, and patients was employed to build the ISC to ensure feasibility and to foster stakeholder buy-in [29]. This work resulted in the ISC model (Figure 1 and Box 1).

Box 1 **ISC model.**

The ISC model includes three groups: the Steering Committee, the Scientific Advisory Board, and the Working Group.

**The Steering Committee**

*Membership*: twenty-three leaders from various health care communities (i.e., representing sectors across the care continuum, provincial quality improvement, health system innovation, administration and operations, indigenous health, patient partners, and provincial health data management).

*Role*: identify and compile ongoing pragmatic issues faced by healthcare teams tasked with implementing various forms of change (e.g., interventions, initiatives, processes, practices, and policies).

**The Scientific Advisory Board**

*Membership*: twelve purposefully selected international implementation science leaders.

*Role*: (a) review the issues identified by the Steering Committee and identify areas that remain understudied in the broader implementation field, and (b) provide research recommendations to help build knowledge of how to respond to widespread barriers that hinder improvement in Alberta.

**The Working Group**

*Membership*: twelve health system and academic scientists who provide practical and scientific implementation expertise.

*Role*: (a) lead stakeholder engagement and facilitate embedded implementation research partnerships, and (b) collect results from embedded IS research and format for use by Alberta’s broader implementation communities.

## 2. Materials and Methods

**Figure 2:**
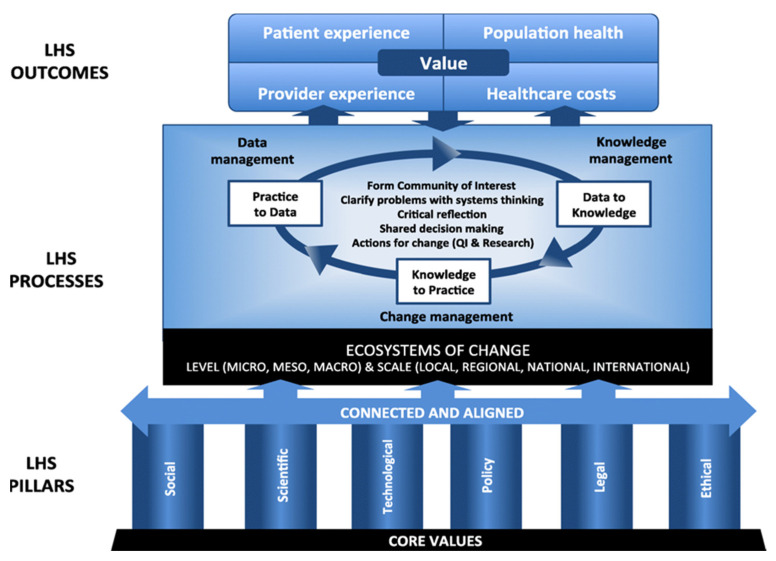
Learning health system conceptual framework [12].

Our team conducted a retrospective evaluation of the ISC planning outcomes to address our research aim and objectives. Our evaluation consisted of (a) a conceptual mapping of the ISC model and (b) a document analysis to compare alignment of the ISC with Menear et al.’s learning health system conceptual framework [12] (Figure 2).

### 2.1. Conceptual Mapping

We conceptually mapped and conducted a comparative analysis of the functions and processes of the ISC model (Figure 1) with Menear et al.’s learning health system conceptual framework [12] (Figure 2) developed in Canada. We compared the ISC model with Menear et al.’s overarching constructs of a learning health system: (a) core values; (b) pillars (i.e., infrastructure and resources); (c) connecting and aligning functions; (d) learning processes; and (e) outcomes [12].

### 2.2. Document Analysis

A document analysis of 148 documents (internal meeting notes, stakeholder event documentation, and stakeholder meeting documentation) from the ISC planning and co-design phases (January 2018–August 2021) was performed. We extracted descriptions of ISC work processes (i.e., how the work is to be completed) and purposes (i.e., the underlying logic of the proposed processes, or why the organizers think this structure will work) to identify alignments and departures from Menear et al.’s conceptual framework [12]. We used these forms of data to evaluate the potential for the ISC to strengthen Alberta’s learning health system and in turn perform as a whole system approach to improvement.

Documents were organized in NVivo12 qualitative data analysis software. We used the ‘exact matches’ text search query function in NVivo to search the document dataset for the terms “learning health system*” and LHS to collect conversations and decisions regarding learning health systems. Further synonym text searches were performed using the terms goal, process, role, and work to collect examples of key considerations for co-designing the ISC in line with evidence-based learning health system constructs. Interpretation of the document analysis was verified with ISC initiative leads (T.W., D.J.) and staff (D.T., G.Z.).

## 3. Results

Of the 148 documents screened, 351 references to either “learning health systems” or LHS were found across 70 documents. These documents (*n* = 70) were scanned for relevance. Duplicates (*n* = 3) and irrelevant documents (e.g., those that mention the learning health system team but nothing more) (*n* = 24) were removed from the dataset, leaving 43 relevant documents to be analyzed. Subsequent synonym searches within the final subset yield results from the terms goal (*n* = 15), process (*n* = 23), role (*n* = 13), and work (*n* = 38). From our conceptual mapping and document analysis, we found clear alignments between the ISC model and Menear et al.’s learning health system conceptual framework constructs [12]: (a) core values; (b) learning health system pillars (i.e., infrastructure and resources); (c) connecting and aligning functions; (d) learning processes; and (e) outcomes. Below, we present a summary of our findings (Table 1), followed by details and examples pulled from our document analysis.

### 3.1. Core Values

The ISC team members used a co-design approach throughout their planning, resulting in a multi-stakeholder governance structure. This structure provides regular opportunities for further co-creation, demonstrating inclusivity, shared leadership, and accessibility, which are learning health system core values. Many of the ISC members have organizational mandates to address disparities in equity, diversity, and inclusion in health care. Our findings identified evidence of the ISC’s meaningful efforts to engage diverse stakeholders in its design, planning, and implementation, which aligns with the core values of Menear’s framework.

### 3.2. Infrastructure and Resources

The resulting ISC model provides numerous infrastructure and resource support in line with four of the six categorizations of Menear’s conceptual framework [12] (social, scientific, technological, and policy). We describe each of these categories below.

Social. The ISC has multi-stakeholder membership and partnerships. The partnerships ensure that the ISC is supporting priorities already established within the health system. Our conceptual mapping identified that establishing a mechanism to communicate implementation research findings across the system is a priority infrastructure needed for the ISC. The purpose of this mechanism is to ensure effective communication across health care sectors and academia.

Scientific. To ensure scientific infrastructure and resources, the ISC used a rubric of competencies required of IS lab scientists [32] (Table 2). The goal of these competencies is to build the individual and collective expertise needed to enable a learning health system. In the ISC model planning, the team engaged individuals with several of these competencies and embedded researchers. Our document analysis showed that many ISC Scientific Advisory Board members were recruited from outside of Alberta due to a lack of local competencies. In an effort to build implementation science capacity locally in Alberta, the ISC is engaged in training programs (i.e., the CIHR Health System Impact Fellowship) and educational activities (i.e., Community of Practice).

Technological. From our analysis, we identified considerations on the role of the ISC in advancing the use of data from Alberta Health Services’ electronic health record infrastructure (EPIC) system to accelerate a learning health system. Currently, the ISC Steering Committee and Scientific Advisory Board include members who are data science experts and have been directly involved with the implementation of the EPIC system. This expertise provides the skill and knowledge for the ISC to support innovation projects focused on creating guidance for the use of EPIC. This will enable high-quality clinical data to be collected, aggregated, analyzed, and acted on.

Policy. We identified that the shared accountability governance structure is a critical characteristic of the ISC model that aligns with a learning health system. This governance structure is largely participatory and inclusive of trans-sectoral health care and research leaders. The ISC shared leadership and accountability structures align with Menear’s conceptual framework. The ISC facilitation is co-led by local health system leaders (T.W., D.J.), who also hold academic appointments. This structure is intended to ensure responsiveness to evolving needs of the learning health system through collaboration.

### 3.3. Learning Health System Learning Processes

To accelerate a learning health system, the ISC model is designed to support existing innovation projects by embedding implementation science research questions into projects seeking funding or that are already underway. This design allows the ISC to integrate existing resources and knowledge, promoting further system integration and increasing the efficiency and effectiveness of implementation-focused learning processes. The ISC model shows that the specific learning cycle of the ISC follows the practice-to-data, data-to-knowledge, knowledge-to-practice cycle described in existing learning health system literature [12].

### 3.4. Outcomes

The documents included in our analysis repeatedly showed the ISC’s commitment to advancing the quadruple aim. This is evidenced in the prioritization principles presented below (Table 3), which will help guide decisions about which health system innovations will be supported by the ISC.

These goals are also present in a key concept document used to engage new stakeholders throughout the planning phase. This document states that “implementation science can enable improvements in alignment with the wide scope of learning health system aims (i.e., to improve health outcomes, care experiences, and health delivery costs)”. The ISC is also currently conducting research to build an impact framework and evaluation plan, recognizing the importance of rigorously assessing how the ISC impacts patient, provider and system outcomes over time.

### 3.5. Key Activities for Co-Designing an IS Lab

**Figure 3:**
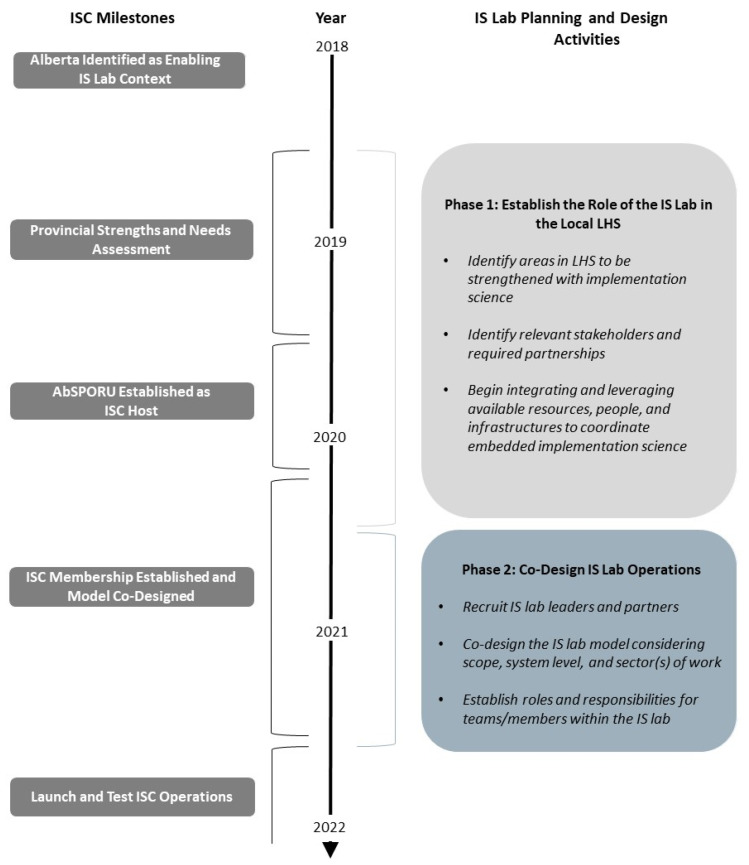
Timing and key activities for co-designing an IS lab.

Our document analysis provided insights into some of the key activities during the co-design of an IS lab. These insights fell into two broad categories: (1) understanding the role of the IS lab within the context of the wider learning health system and (2) establishing IS lab processes needed to achieve that role (Figure 3).

#### 3.5.1. Understanding the Role of the IS Lab within the Context of the Wider Learning Health System

Early in ISC initiative planning (Phase 1), the initiative organizers hosted multi-stakeholder engagement events to identify if and how an IS lab could strengthen Alberta’s existing learning health system. These groups identified trans-sectoral communication as a key barrier to effective implementation and subsequent scale, spread, and sustainment of innovations. Furthermore, rigour of implementation planning and evaluation varied. Without standard measures, teams could not tell if innovation failures were attributable to intervention or implementation issues. Conversely, when innovations succeeded, documentation was not strong enough to inform scale and spread or to provide lessons for innovations in other contexts. The main goal of the ISC became to successfully integrate implementation science into healthcare improvement through standardization of implementation and evaluation at a provincial scale. One such example of this articulation was found in an event summary from June 2021 stating that: “By using implementation science principles and theories to support a broad range of improvement initiatives (i.e., both implementation and quality improvement projects), the ISC will be uniquely and strategically positioned to generate robust, generalizable knowledge to benefit learning health system stakeholder communities”.

#### 3.5.2. Establishing IS Lab Processes

From our document analysis, we identified a distinct shift in focus for Phase 2 of planning. Questions arose of how to complete work within the resource and capacity limitations of the ISC staff (*n* = 5) and the time constraints of members who all have leadership roles in the health system or academia. After articulating the goals of the ISC, extensive stakeholder engagement, through monthly Steering Committee meetings, was carried out to continually assess and refine the ISC implementation planning. The Steering Committee meeting discussions were bolstered by a series of smaller engagement activities with health system administration and operations leaders, as well as implementation and system scientists. The Steering Committee identified missing stakeholder groups and recruited new members as discussions of meeting learning health system needs evolved.

A key consideration that emerged through these engagement activities was the accountability structures and measures for the ISC model (i.e., who would be responsible for the ISC and its governance structure, should it be managed by academia or the health system). In Alberta’s health context, it became clear that creating a collaborative among health system sectors and academic institutions was necessary to avoid exacerbating conditions that cause silos and fragmented implementation research in the system. This approach was made explicit in a concept document that was used for engagement with external stakeholders. An example is outlined in Box 2.

Box 2 **Evidence to support creating a collaborative.**

*“There is a broad stakeholder community that has contributed to many achievements to improve the healthcare system. Uniting these improvement efforts through collaboration is required to enable a Learning Health System. Having a single healthcare system for the entire province creates the potential for coordinated province-wide action and collaboration between the health system and implementation scientists. However, the siloes within which health care practitioners, policymakers and health researchers operate, hinder system-level collaborations to study which implementation methods work (or not), in which Albertan contexts. There is currently no existing mechanism to bring these fields together in a way that allows Alberta to create, synthesize, and share generalizable, real-world, evidence-based guidance for implementation in Albertan contexts. This ISC can provide this missing mechanism.”*

Another key consideration for the ISC was on prioritization principles and processes. To guide ISC work, the member groups worked together to develop prioritization principles (Table 3). The prioritization principles were drafted using research priorities found in IS literature and the organizational mandates of the members. Through collaborative review the ISC distinguished screening criteria from prioritization principles. Together, these considerations will help guide decisions about which health system innovations will be supported by the ISC.

## 4. Discussion

Our findings demonstrate the potential of IS labs as an approach to integrating implementation science and practice, to better enable a learning health system to achieve whole system improvement. Healthcare often exists in sectoral silos, conducting health innovation projects independent from one another. Moreover, it is common to experience continuous cycles of pilot innovation projects, ultimately perpetuating research waste and inefficiency across health systems and a failure to develop a whole system approach to improvement [33,34]. Unlike traditional paradigms of implementation and healthcare improvement, where research is “pushed” from researchers to health systems [11], the ISC model was co-designed in a way that enables a collaborative, whole system approach to improvement and implementation through integration of science and practice. The ISC model is underpinned by shared governance and accountability structures, multi-stakeholder involvement, trans-sectoral engagement and partnerships, and an integrated, adaptive approach to improvement. These features are fundamental to achieving a whole system approach to improvement [12].

As highlighted by others, IS labs can enable learning health systems by providing an increased likelihood of applied evidence for health system improvement [21,22,23,35]. They also support sustained implementation success through multi-stakeholder partner engagement and embedded implementation science and practice to achieve long-term impact [21,35]. Our conceptual mapping with Menear’s framework [12] highlighted that the ISC has undertaken many activities of engagement, which in turn will facilitate successful implementation. The ISC embodies a multi-stakeholder governance structure and processes based on the principles of fairness, equity, and transparency. The ISC has built and leveraged cross-sectoral research partnerships to identify and address cross-sectoral implementation barriers through regular ISC member group meetings and outreach engagement activities with policy makers, researchers, and operation teams. A system-wide communication process is currently being developed for collecting ISC research results and sharing them back to multi-stakeholders involved in implementation and healthcare improvement.

IS labs are designed to be collaborative in nature, enabling reciprocal and mutually beneficial relationships and outcomes for researchers and healthcare system stakeholder partners [22]. Our analysis identified key activities and considerations to ensure a collaborative and mutually beneficial IS lab, such as developing and engaging cross-sectoral representation of people working with different communities, conditions, and at different levels of the health system. The ongoing engagement activities and considerations during the ISC co-design and planning helped to identify missing stakeholders, system needs, and leverage points for integration. Our findings highlighted that the ISC model has international implementation science representation in its members; however, this also uncovers implementation science capacity gaps in Alberta.

IS labs offer greater opportunities to understand, through science, how and why implementation works well or not and how to improve innovation uptake, scale, spread and sustainability through a collaborative approach. Ultimately, IS labs offer a potential mechanism to achieve the greatest impact with healthcare improvement efforts on patients, providers, care, and health systems [21,22,23,35,36,37,38]. Implementation science and practice integration focused on foundational assets and infrastructure (e.g., data systems, common measures, and networks) that can enable learning in real-time and continuous improvement of policies and programs [39,40]. Our conceptual mapping and document analysis findings illustrate how the ISC model has considered and leveraged existing assets and infrastructures to enable a learning health system and support a whole system approach to improvement. It is important to also note that the ISC model is integrating implementation science into an established learning health system and that other IS labs that are transforming a health system may have different considerations—ethics, legal, and policy.

Grimshaw et al. [21] detailed the importance of stability in healthcare system partners, team composition, identifying shared priorities, aligning timelines and activities, ethical considerations, and funding for the establishment of an IS lab. These considerations provide useful guidance for other stakeholders and organizations considering the development, implementation, and long-term sustainability of an IS lab to enable learning health systems. The ISC model illustrates the potential of IS labs to achieve cumulative knowledge for implementation and healthcare improvement. The ISC’s shared governance and multi-stakeholder partnerships across sectors (e.g., academia, acute healthcare, primary healthcare, LTC) have the potential to reduce healthcare and research waste and improve system-wide learning [21].

### Future Research

Our findings highlight the potential roles of IS labs in supporting learning health systems. For example, the ISC can perform key connection, integration, and communication functions at a systems level. This article presents the learning health system constructs and contextual factors that led to the decisions to take on these functions. Organizers in other contexts will need to conduct activities to assess the state of their own learning health systems and the needs of their stakeholders. Follow-up studies by the authors of this article are underway to create evidence-based guidance for IS lab planning, implementation, operations, adaptation, and how to build and sustain the partnerships involved in all phases. ISC organizers will also conduct a rigorous research impact assessment to strengthen the literature on how IS labs can impact learning health system outcomes [41]. Such research is crucial to understand their value and benefits for patients, stakeholders, research, and health system outcomes.

## 5. Limitations

This study comes from a Canadian perspective, and similar methods may need to be tested in other contexts to assess the fit and value of IS labs in other learning health system contexts (e.g., American health management organizations that are privately funded).

## 6. Conclusions

Our conceptual mapping and document analysis demonstrate the alignment between the ISC model and Menear’s learning health system conceptual framework. Our findings reveal how and in what ways IS labs represent the core values, infrastructure and resources of a learning health system. The next steps of the ISC model are to develop capacity and sustainability plans, with the understanding that meaningful whole system improvement through IS labs is unachievable without long-term sustained effort.

## Figures and Tables

**Table 1:** Summary of comparative analysis of the ISC model to Menear’s conceptual framework.

Menear’s Framework	ISC Model
Core values	
Accessibility	All co-design and operations decisions of the ISC are driven by the Steering Committee, which is guided by multi-stakeholder participatory leadership, inclusivity, and shared accountability. The ISC ensures scientific integrity by incorporating international implementation science experts with varying skillset across all groups within the ISC model (Table 2). The ISC strives for accessibility, adaptability, and value in healthcare through the multi-stakeholder governance structure and processes by which the Steering Committee monitors the work of the ISC and ensures that it meets the priorities of the system. The principles that guide ISC work (see Table 3) include fairness, equity, and transparency.
Adaptability	
Cooperative and participatory leadership	
Shared accountability	
Inclusiveness	
Person focused	
Privacy	
Scientific integrity	
Transparency	
Equity	
Fairness	
Solidarity	
Value in healthcare	
**Infrastructure and resources**	
Social	The ISC has built and leveraged cross-sectoral research partnerships to identify and address cross-sectoral implementation barriers through regular ISC member group meetings and outreach engagement activities with policy makers, researchers, and operation teams. A system-wide communication process is currently being developed for collecting ISC research results and sharing them back to multi-stakeholders involved in implementation and healthcare improvement work.
Scientific	The ISC has international implementation science expertise from Canada, the United Kingdom, and the United States as well as Alberta-based implementation-relevant researchers (e.g., data analysts, health economists, community-based researchers, and system scientists). The ISC hosts monthly, expert-led community of practice seminars to build IS capacity in ISC members and stakeholders. To build embedded research capacity and implement scientific expertise, the ISC participates in the Health System Impact Fellowship funded by the Canadian Institutes for Health Research [30,31].
Technological	All ISC member groups have individuals with expertise in the local electronic health record informatics systems. This is crucial for designing embedded implementation research that relies on various electronic health records for data collection.
Policy	The structured planning of ISC innovation projects aims to standardize implementation and evaluation processes system wide, in turn creating evidence to inform policy decisions.
Legal	The ISC works within Alberta’s existing health system legal structures, processes and policies to strengthen the evolving provincial learning health system.
Ethical	The ISC operates within ethical structures and processes established by Alberta Health and Alberta Health Services and partnered academic institutions.
**Connecting and aligning functions**	
The distinct learning health system	The ISC aims to integrate Alberta’s existing learning health system pillars (e.g., electronic medical records, implementation science expertise, and innovation teams) to study implementation methods and build principles of implementation that support more effective and efficient implementation of innovation province wide (learning health system processes). More effective implementation will bring health care innovations to patients and providers faster than previously possible. This is in line with learning health system goals of advancing the quadruple aim.
infrastructures and resources must be connected and aligned to support learning and advance goals	
**Learning processes**	
The system creates new knowledge from practice-based data, uses this knowledge to inform improvements, documents improvements to create data	The ISC aims to study cross-sectoral implementation to accelerate effective spread, scale, and sustainment of innovations across the health system, situating its work in the meso and macro levels of change. The ISC aims to leverage implementation science expertise (i.e., the Scientific Advisory Board) to provide expert input to support embedded implementation science into existing health system innovation projects. The ISC Working Group aims to synthesize and distribute implementation science research findings to the Alberta implementation community to accelerate effective implementation, scale, spread, and sustainment of innovations. The ISC aims to ensure the principles for implementation are feasible for Alberta’s innovation ecosystem through health system policy-maker participation on the ISC Steering Committee.
**Outcomes**	
Quadruple Aim [16] (i.e., improve patient experience, improve provider experience, improve patient outcomes, increase value for money)	The explicit mandate of the ISC is to support innovation projects that advance the quadruple aim in Alberta. The ISC is building an evaluation framework to guide impact assessment. There is explicit mention of the Quadruple Aim in the principles that guide the work of the ISC (Table 3).

**Table 2:** Competencies of IS lab scientists.

Skillset
Improvement and Implementation Science
Data Science and Informatics
Real-World Research
Research Question and Evidence
System Science
Engagement, Leadership, and Research Management
Ethics of Research and Implementation in Health Systems
Health Economics

**Table 3:** ISC prioritization principles.

Screening Criteria for Potential Priority Issues—If the Issue or Question Cannot Meet All Three Screening Criteria, It Will Not Be Considered for ISC Support.
Is this question answerable through implementation science informed research?
2 Will answering this implementation science question advance our system towards the quadruple aim (i.e., improve patient experiences, improve provider experiences, improve patient/population health outcomes, and ensure value for money) and/or towards improving equity, diversity, and inclusion in health care delivery?
3 Has this question been co-designed and/or endorsed by patients, other stakeholders, and sector(s) affected by the implementation?
**Principles to Prioritize Implementation Issues Supported by the ISC**
4 Would the solution to this question create new knowledge to support a practical approach to implementation science and/or practice?
5 Does this question explore and address barriers, context, and fidelity within implementation; scale, spread; and/or sustainability?
6 Does answering this question resolve or inform implementation barriers that could apply across multiple sectors on the care continuum?
7 Would an answer to this question be of value to decision makers/policy makers and influence either healthcare investment or new policy?

## Data Availability

The documents that informed this study can be made available upon request through contacting the Alberta SPOR SUPPORT Unit Learning Health System Team (stephaniebrooks@ualberta.ca).
